# Head-Mounted Display Virtual Reality for Cognitive and Physical Function in Older Adults: A Scoping Review

**DOI:** 10.1093/geroni/igaf122.3950

**Published:** 2025-12-31

**Authors:** Ferdinand Delgado, Sanghee Moon

**Affiliations:** University of New Hampshire, Durham, New Hampshire, United States; University of New Hampshire, Durham, New Hampshire, United States

## Abstract

Head-mounted display virtual reality (HMD-VR) shows promise for supporting cognitive and physical function in older adults, yet researchers face challenges identifying appropriate applications for interventions. This scoping review systematically mapped HMD-VR applications used in research with older adults, creating a comprehensive catalog to guide future interventions, support new development, and help clinicians identify appropriate interventions. Following PRISMA-ScR guidelines, we searched PubMed, CINAHL, and Web of Science using terms related to VR technology, older adults, and cognitive/physical function. Twelve studies published 2009-2024 were included, encompassing 809 participants aged 55+ years (average age range: 67.9 to 85.9 years). We identified 32 distinct HMD-VR applications across platforms, including Oculus Quest (n = 6 studies), HTC VIVE (n = 3), and others (n = 3). Custom-developed applications dominated the field (87.5%, n = 28 applications), with only three commercially available applications used: Wander, YouTube VR, and Maze Walk, with one unknown. From the identified applications, attention was the most frequently targeted cognitive domain (n = 24), followed by executive function (n = 17), visuospatial function (n = 12), and memory (n = 9). Upper limb movement was the primary physical target (n = 15), followed by lower limb movement (n = 12), and balance training (n = 9). Eleven out of twelve studies reported positive cognitive outcomes, while six out of eight studies showed improved physical outcomes. This mapping reveals that researchers predominantly create specialized VR, suggesting the need for more accessible, evidence-based applications. Our scoping review provides researchers and clinicians with potential application options for designing HMD-VR interventions targeting cognitive and physical function in older adult populations.

